# Sight

**Published:** 1874-03

**Authors:** 


HALL'S

### JOURNAL OF HEALTH

AND

MISCELLANY.

VOL. XXI.—MARCH, 1874.—No. III.

### SIGHT.

If a pebble is thrown in the water a wave is made; another and another follows on with a rapidity proportioned to the greater disturbance of the fluid. There is an invisible fluid between us and the sun, which, if set in motion, has waves of its own, moving one after another with almost inconceivable rapidity, a hundred and eighty thousand miles in a second of time, some of them, and striking against the eye they produce what we call light or color; the difference in the colors of the light being according to the speed and number of these waves or vibrations—the length of these waves being from the twenty-five to the seventy-five thousandth part of an inch. If these waves have a much greater or less velocity, they convey no impressions on the eye of anything being present—it does not see—they are invisible. There is a fluid not as thin as ether, not as thick as water, that is air—its waves, its vibrations striking against the ear, or rather the drum or tympanum of the ear, cause the sensation of sound. Although the eye may not be sensitive enough to perceive impressions from infinitessimally small waves of ether, as the vibration of air may be too minute to convey to the ear any sound, yet a long piece of wood or steel, or a trumpet shaped apparatus, will make a sound audible which was not so before. So in the case of ethereal vibrations—they may not have force enough to make themselves perceived to the eye, and are, therefore, invisible; yet a photographic plate may be so delicately prepared as to give an impression of something which the naked eye could not perceive. In another direction a spy-glass or a common pair of spectacles, if they do not bring invisible objects to us, make objects almost invisible quite clear, distinct, plain. Hence, if there is such a thing as a spirit or a ghost, a little less impalpable than air, its photograph could be taken. Hence, a photograph not visible to the naked eye, if placed near a sitter, would be impressed on the photographer's plate with sufficient distinctness to be seen. Hence the mystery of having the likeness of departed friends appear on the same plate with the sitter. The likeness resembling the departed depends on the vividness of the imagination of the sitter or of simple fortuitous coincidence—just as in the millions of dreams now and then, with the aid of a little stretching of the imagination, one is verified. A more direct method is to paint a portrait of the person desired to be seen, on the screen behind the sitter, with a solution of common sulphate of quinine, which, on drying, becomes invisible to the common eye, but still emits a kind of phosphorescent light, sufficient to make a faint or ghostly impression on the delicate photographic plate, giving it a spiritual, impalpable appearance, well calculated to awe the superstitious and uninformed.

